# A Useful Method to Provide Infectious and Cultivable In Vitro Naked Viral Particles of Hepatitis A Virus

**DOI:** 10.3390/v16091360

**Published:** 2024-08-26

**Authors:** Gwenaëlle Verbrugghe, Chloé Soudan-Foulques, Audrey Fraisse, Prunelle Waldman Vigne, Sylvie Perelle, Fatou-Toutie Ndoye, Sandra Martin-Latil

**Affiliations:** 1 Université Paris-Saclay, INRAE, UR FRISE, 92160 Antony, France; fatou-toutie.ndoye@inrae.fr; 2 ANSES, Laboratory for Food Safety, UVE, 94700 Maisons-Alfort, France; audrey.fraisse@anses.fr (A.F.); sylvie.perelle@anses.fr (S.P.); 3 ANSES, Animal Health Laboratory, UMR1161 Virology, INRAe, Anses, ENVA, 94700 Maisons-Alfort, France; chloe.soudan@vet-alfort.fr

**Keywords:** hepatitis A virus, delipidation, replication kinetics

## Abstract

Hepatitis A virus (HAV) is an enteric virus mainly transmitted by the faecal–oral route. Belonging to the *Picornaviridae* family, HAV was first described as small naked particles, like all viruses of this family. However, for about a decade, it was demonstrated that HAV particles can exist surrounded by a lipid bilayer. This type of particle, called enveloped HAV (eHAV), acquires its lipid bilayer by hijacking a part of cell membranes during the virion egress in the last steps of the viral cycle. In vitro culture systems produce mainly eHAV, and so, to date, most of the studies on HAV have been carried out using this type of viral particle. In this study, a method based on lipid bilayer removal by chemical delipidation is proposed for the production of naked HAV particles. The resulting naked HAV particles conserve their infectivity and are therefore fully cultivable in vitro. By using this method, naked HAV particles can easily be produced in vitro and can be useful to perform further studies such as inactivation processes for the food industry, as HAV is a main concern for food safety.

## 1. Introduction

Hepatitis A virus (HAV) belongs to the *Hepatovirus* genus in the *Picornaviridae* family. Its single-stranded, positive-sense RNA genome contains an open reading frame (ORF) coding for a polyprotein cleaved into structural and non-structural proteins including the RNA-dependent RNA polymerase (RdRp) [1]. HAV is a small particle of around 27 nm, firstly described as non-enveloped, or naked, as are all *Picornaviridae* family members [2]. Nevertheless, HAV particles have also been described as enveloped. Such particles, called eHAV, acquire their lipid bilayer by hijacking host cell membranes during the last step of the viral cycle, i.e., virus release [3]. eHAV differs from conventional enveloped viruses because of the lack of encoded viral glycoproteins in the envelope and is in that way considered quasi-enveloped. However, the eHAV quasi-envelope also contains cellular membrane proteins acquired during the egress of viral particles from infected cells. eHAV has a density and size similar to cellular exosomes, and nHAV has been described as having a greater density than eHAV [4].

Even if the mechanisms involved in the virus spreading to the liver remain unknown, HAV enters an organism through the digestive tract [5]. HAV is mainly replicated in hepatocytes, epithelial polarised cells [6]. eHAV particles egressing from hepatocytes by the basolateral membranes reach the bloodstream in a quasi-enveloped form, and those released from the apical membrane reach biliary canaliculi. Due to the detergent properties of bile, the quasi-envelope is removed and HAV particles reach the gastrointestinal tract in a naked form [4]. Thus, eHAV circulates in the bloodstream of infected people, while naked HAV (nHAV) is excreted in faeces [7]. Different biomolecules or chemical agents have been used to produce naked HAV particles from quasi-enveloped particles such as chenodeoxycholic acid (CDCA), taurocholic acid (TCA) [4], or 1% IGEPAL ^TM^ CA-630 [3]. Other treatments have been tested such as 5% Tween-20, 10% chloroform, 0.1% NP-40 + 0.1% 2-MercaptoEthanol (2-ME), or 0.1% NP-40 + 0.1% pronase E [8] for hepatitis E virus (HEV) quasi-envelope removal, since HEV, like HAV, exists as naked and quasi-enveloped particles [9].

HAV infections mainly occur by the faecal–oral route, either through direct contact or by the consumption of contaminated food or water [10]. Since nHAV is released in the stools of infected patients, these naked viral particles are found in wastewater and thus circulate and persist in the environment. Studies on the persistence and inactivation of HAV are usually carried out using clarified supernatants from infected cells. Even if the observations highlighted by these studies have given better insights into HAV behaviours on several foods, viral stocks produced by in vitro cell culture systems are predominantly composed of quasi-enveloped particles [11,12,13,14,15,16]. To date, there is still a lack of data on purified nHAV to study its persistence in the environment and its resistance to inactivation processes. Moreover, extrapolation to studies carried out with eHAV may not be appropriate without any comparative studies of both types of particles. Naked particles of HAV have been produced or isolated to study their entry mechanism [17,18] or their replication in cells [4], but to the best of our knowledge, replication kinetics of nHAV and eHAV have not been established and compared yet.

The aims of this study were to propose a delipidation method to remove the quasi-envelope of eHAV for the production of naked HAV particles and to subsequently compare the replication kinetics of nHAV with those of eHAV.

## 2. Materials and Methods

### 2.1. Cell Line and Culture Conditions

FRhK-4 cells (Fetal Rhesus Monkey Kidney, CRL-1688, ATCC) were cultivated in a growth medium containing DMEM-Glutamax (Dulbecco’s Modified Eagle’s Medium) supplemented with 1% non-essential amino acids, 1 mM sodium pyruvate, and 10% fetal calf serum (FCS). Cells were incubated at 37 °C with 5% CO_2_. Cell maintenance was carried out each week by detaching and individualising FRhK-4 cells by using 0.05% trypsin and 0.05% EDTA and then seeding cells in new culture flasks (Falcon™) at a ratio of 1:4. All components used for cell culture were purchased from Fisher Scientific (Illkirch, France).

### 2.2. HAV Amplification by Cell Culture

FRhK-4 cells were infected by the cytopathic culture-adapted HM175/18f strain of HAV (CR-1402, ATCC) at an M.O.I. (Multiplicity Of Infection) of 0.01 in FCS-free culture medium. After 6 or 7 days of infection, when at least 90% of the cytopathic effect could be observed on the cell monolayers, cell flasks were frozen at −80 °C and then thawed in a 37 °C water bath. The cell culture supernatant was clarified at 5000× *g* for 15 min at 4 °C to remove cell debris.

### 2.3. Viral Particle Purification

**Figure 1:**
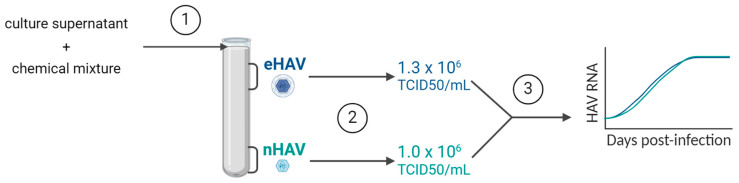
Experimental overflow. The culture supernatants were chemically delipidated ➀ and then ultracentrifugated to separate eHAV and nHAV. Each type of particle was individually withdrawn from the gradient, purified, and titrated by a TCID50 assay ➁ to produce viral stocks in order to perform replication kinetics ➂.

Density gradients were prepared in Polyallomer Thick Wall (13.5 mL) centrifuge tubes (Beckman Coulter, Roissy-en-France, France) by successively and gently adding 2.3 mL of 60% (*wt*/*wt*) and 1.7 mL of 50%, 40%, 30%, 20%, and 10% (*wt*/*wt*) sucrose solutions (total gradient volume, 10.8 mL) prepared with a pH 7.5 buffer containing 0.01 M Tris-HCl, 1 mM EDTA, and 150 mM NaCl (Sigma-Aldrich, Saint-Quentin-Fallavier, France). The tubes were stored overnight at 5 °C to allow for continuous gradient formation. After ultracentrifugation, as described in the following section, twelve fractions of 1 mL were collected and the density of each was determined using a refractometer (Bellingham + Stanley, Weilheim, Germany). Then, viral particles were purified as described in the following sections (Figure 1).

#### 2.3.1. eHAV Stock

The clarified supernatants (1.4 mL) were loaded on top of continuous sucrose gra-dients. Viral particles were ultracentrifuged at 191,500× *g* with the MLA-55 fixed-angle rotor (Beckman Coulter) for 2.5 h at 4 °C. The fractions containing eHAV were retrieved (fractions 5, 6, and 7) and diluted at 1:10 in PBS (Phosphate-Buffered Saline, Gibco). eHAV was then purified and concentrated by using ultrafiltration with Ultracel Regenerated Cellulose (30 kDa MWCO) Ultra-15 units (Merck, Fontenay-sous-Bois, France) at 5000× *g* for 5 min at 4 °C. The concentrated eHAV stock was aliquoted and stored at −80 °C.

#### 2.3.2. nHAV Stock

The clarified supernatants (1.4 mL) were treated with three different chemical mixtures: 5% Tween-20 with 0.1% 2-MercaptoEthanol (2-ME), 0.1% IGEPAL ^TM^ CA-630 with 0.1% 2-ME, or 0.1% IGEPAL ^TM^ CA-630 in FCS-free culture medium. After incubation at 37 °C with soft agitation at 160 rpm for 2 h, each mixture was placed on top of continuous sucrose gradients and ultracentrifuged at 191,500× *g* for 2.5 h at 4 °C. Twelve 1 mL fractions were successively collected. HAV RNA was then extracted from 500 µL of each fraction and quantified by RT-qPCR, as described below.

For further purification of the nHAV stock following delipidation using the treatment with 5% Tween-20 and 0.1% 2-ME, the fractions containing nHAV were withdrawn (fractions 10, 11, and 12) and diluted at 1:10 in PBS (Phosphate-Buffered Saline, Fisher Scientific). nHAV was then purified and concentrated by using ultrafiltration with Ultracel Regenerated Cellulose (30 kDa MWCO) Ultra-15 units (Merck) at 5000× *g* for 5 min at 4 °C. The concentrated nHAV stock was aliquoted and stored at −80 °C.

### 2.4. Infectious Titration of HAV

Viral titration was achieved by using a Real-Time Cell Analysis (RTCA) assay using the viral-induced cell impedance drop as a readout of cell infection [19]. FRhK-4 cells were seeded in 96-well E-plates (xCELLigence RTCA E-plate 96, Agilent, Les Ulis, France) with a density of 10^4^ cells per well. After 48 h, cell monolayers were washed with 200 µL of FCS-free medium and infected with 100 µL of serial dilutions of the viral stocks in FCS-free culture medium. Eight wells were inoculated with each dilution. After 2 h of viral adsorption, 100 µL of 4% FCS culture medium was added to each well (2% FCS final concentration). Cell impedance was recorded for each well in real time by using the MP xCELLigence system (Agilent) and a drop of cell impedance in infected cell monolayers was considered positive for HAV infection. Infectious titres were calculated after 14 days of infection using the Spearman–Kärber calculation method and were 1.3 × 10^6^ TCID_50_/mL and 1.0 × 10^6^ TCID_50_/mL for eHAV and nHAV, respectively.

### 2.5. Virus Growth Kinetics

FRhK-4 cells were seeded in 96-well plates at a density of 10^4^ cells per well in 200 µL of growth medium. After 48 h of incubation at 37 °C and 5% CO_2_, cell monolayers were washed once and infected at an M.O.I. of 1 with eHAV or nHAV in an FCS-free culture medium. After 1.5 h of viral absorption, the viral inoculum was removed and the cell mo-nolayers were washed twice with 200 µL of FCS-free culture medium. Culture medium supplemented with 2% FCS was then added to each well and the cells were incubated for 7 days at 37 °C with 5% CO_2_. At days 0, 1, 2, 3, 4, and 7 post-infection, culture supernatants were collected and clarified at 500× *g* for 5 min at 4 °C. The cells were washed twice with 100 µL of 0.05% trypsin and 0.05% EDTA, then harvested by trypsinisation for 5 min at 37 °C, and pelleted at 500× *g* for 5 min at 4 °C. The clarified supernatants and pelleted cells were stored at −20 °C.

### 2.6. Nucleic Acid Extraction and Quantification of HAV Genomes by RT-qPCR

Cell culture supernatants (500 µL), sucrose gradient fractions (500 µL), or cell pellets were mixed with Nuclisens^®^ lysis buffer (3 mL final volume). After 20 min at room temperature, 50 µL Nuclisens^®^ magnetic silica beads were added to each lysed sample. RNA purification and elution in 70 µL Nuclisens^®^ Buffer 3 were achieved by using the automated Nuclisens^®^ EasyMag^TM^ system (Biomérieux, Marcy-l’Étoile, France). Extracted RNAs were stored at −80 °C for further HAV RNA titration.

Viral RNA was quantified by RT-qPCR using the RNA Ultrasense™ one-step quantitative RT-PCR kit (Fisher Scientific). HAV68_5′-TCACCGCCGTTTGCCTAG-3′ forward primer, HAV240_5′-GGGAGCCCTGGAAGAAAG-3′ reverse primer, and HAV150_5′FAM-CCTGAACCTGCAGGAATTAA-3′MGB TaqMan probe [20] were added to the Ultrasense^TM^ reaction mix. A volume of 5 µL of the extracted RNA was mixed into 20 µL of the reaction mix, whose composition is presented in Table 1. The CFX96 Real-Time PCR Detection System (Bio-Rad, Marnes-la-Coquette, France) thermocycler was used with the following cycling parameters: reverse transcription for 1 h at 55 °C, reverse transcriptase inactivation and thermoresistant Taq-polymerase activation for 5 min at 95 °C, then 45 cycles of 15 s at 95 °C, 1 min at 60 °C, and 1 min at 65 °C for cDNA denaturation, hybridisation, and elongation, respectively. At the end of each elongation step, the fluorescence was recorded using the apparatus. Finally, the genomic titre was calculated using a standard curve, from 1 × 10^5^ to 1 × 10^2^ HAV RNA copies per PCR well, of in vitro RNA transcripts of HAV. The quantification limit of the method is 1 × 10^2^ HAV RNA copies per µL [21]. Standard curve determination was performed in duplicate and sample genomic titration was performed once.

### 2.7. Transmission Electron Microscopy (TEM)

Purified eHAV and nHAV were fixated with 2% glutaraldehyde overnight at 5 °C. Viral particles (4 µL) were collected on 200-mesh copper grids and negatively stained with 1% uranyl acetate in H_2_O. eHAV and nHAV were then observed using the Hitachi HT7700 electron microscope at 80 kV (Elexience, Verrières-le-Buisson, France).

### 2.8. Statistical Analysis

A statistical analysis based on the analysis of variance (ANOVA) was performed to assess the difference in significance between the replication kinetics of eHAV and nHAV. A statistical analysis was performed on the quantity of HAV RNA released in the cell culture supernatants or intracellular HAV RNA. The results were considered significant for a *p*-value less than 0.05.

## 3. Results

### 3.1. Selection of the Most Suitable Delipidation Method for Producing Naked HAV

**Figure 2:**
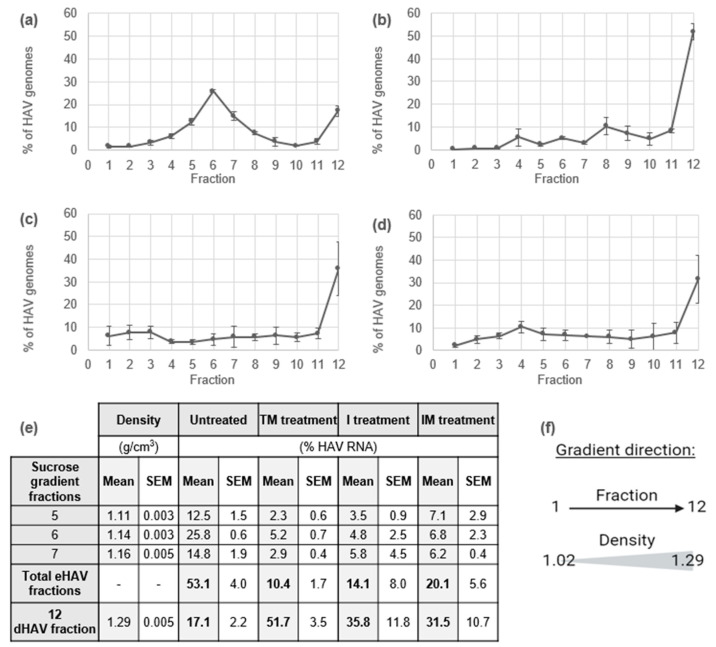
The purification of HAV particles by density gradients. The quantity of the viral genome was determined in each fraction by RT-qPCR. The results are expressed as the mean ± SEM of the HAV RNA percentage in the fraction compared to the total quantity of HAV RNA in the gradient of (**a**) the untreated HAV (cell culture) supernatant, (**b**) the HAV supernatant treated with 5% Tween-20 and 0.1% 2-ME (TM), (**c**) the HAV supernatant treated with 0.1% IGEPAL ^TM^ CA-630 (I), or (**d**) the HAV supernatant treated with 0.1% IGEPAL ^TM^ CA-630 and 0.1% 2-ME (IM). (**e**) The mean density and recovered HAV RNA percentage from gradient fractions containing eHAV or nHAV; (**f**) the gradient direction. (n = 3).

In order to produce naked particles of HAV, different treatments using detergents were assessed regarding their efficiency in removing lipid bilayers from quasi-enveloped particles produced using a cell culture system. Three mixtures were tested for HAV delipidation from HAV produced from FRhK-4 cells: 5% Tween-20 with 0.1% 2-ME (TM), 0.1% IGEPAL ^TM^ CA-630 (I), or 0.1% IGEPAL ^TM^ CA-630 with 0.1% 2-ME (IM). Treated and untreated supernatants were ultracentrifuged on density gradients and the HAV RNA percentage in each fraction was calculated from HAV RNA quantified by RT-qPCR (Figure 2).

First, untreated culture supernatants were analysed to identify both phenotypes of viral particles in the gradients (Figure 2a). Regarding the quantity of HAV RNA quantified in each fraction, two sedimentation areas can be identified in the gradients. First, HAV RNA is mainly found in fractions 5, 6, and 7 (density of 1.11, 1.14, and 1.16 g/cm^3^, respectively), with 12.5, 25.8, and 14.8% of HAV RNA found, respectively (total of 53.1%) (Figure 2e). These fractions correspond to the sedimentation area of eHAV having a lower appa-rent density than nHAV. The second sedimentation area is fraction 12 with a density of 1.29 g/cm^3^, in which 17.1% of HAV RNA is found (Figure 2a,e), which can be attributed to the sedimentation area of nHAV, which has a greater density than eHAV (Figure 2).

Then, to evaluate the delipidation treatment efficiencies, HAV RNA percentages in fractions 5, 6, and 7 for eHAV and in fraction 12 for nHAV were compared with those of the untreated supernatants (Figure 2e). All delipidation treatments lowered the quantity of HAV RNA in the sedimentation area of eHAV compared with untreated supernatants, as the HAV RNA quantity decreased by 42.7% with the TM treatment, by 39.0% with the N treatment, and by 33.0% with the NM treatment. In fraction 12, the HAV RNA quantity increased by 34.6%, 18.7%, and 14.4% with TM, N, and NM treatments, respectively (Figure 2b–e). Regarding these results, the TM treatment was significantly (*t*-test; *p* < 0.05) the most efficient and was selected for nHAV production.

Therefore, eHAV and nHAV were isolated from the gradients, and titrations were then performed to determine the infectivity of the viral stocks produced. The eHAV and nHAV infectious titres were 1.3 × 10^6^ TCID_50_/mL and 1.0 × 10^6^ TCID_50_/mL, respectively, indicating that the delipidation treatment does not impact the infectious properties of the purified nHAV as it can be still quantified using a traditional infectious titration assay.

### 3.2. Verification of HAV Lipid Bilayer Removal by Delipidation Treatment

**Figure 3:**
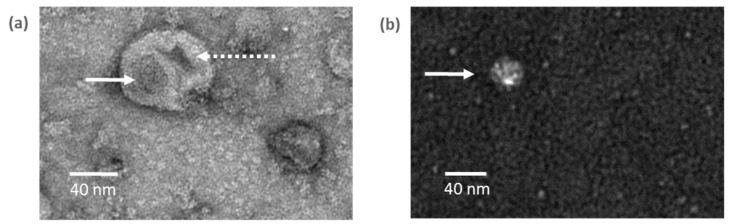
Negatively stained eHAV and nHAV particles visualized by TEM. (**a**) eHAV in untreated supernatants purified by ultracentrifugation. (**b**) nHAV in supernatants treated with 5% Tween-20 and 0.1% 2-ME and purified by ultracentrifugation. Straight arrow: viral particle; dotted arrow: lipid bilayer.

To assess the efficiency of the delipidation process, purified eHAV and nHAV were vi-sualised by TEM after negative staining. As shown in Figure 3, eHAV particles are larger than 40 nm in diameter (Figure 3a). These viral particles (Figure 3a, straight arrow) seem to be surrounded by a lipid bilayer (Figure 3a, dotted arrow). On the contrary, the size of the nHAV particle shown in Figure 3b is less than 40 nm and there is no visible lipid bilayer.

Therefore, the delipidation treatment effectively removed the quasi-envelope of the viral particles to produce naked HAV particles with a diameter of approximately 30 nm and a density in sucrose of approximately 1.29 g/cm^3^.

### 3.3. eHAV and nHAV Replication Kinetics in FRhK-4 Cells

**Figure 4:**
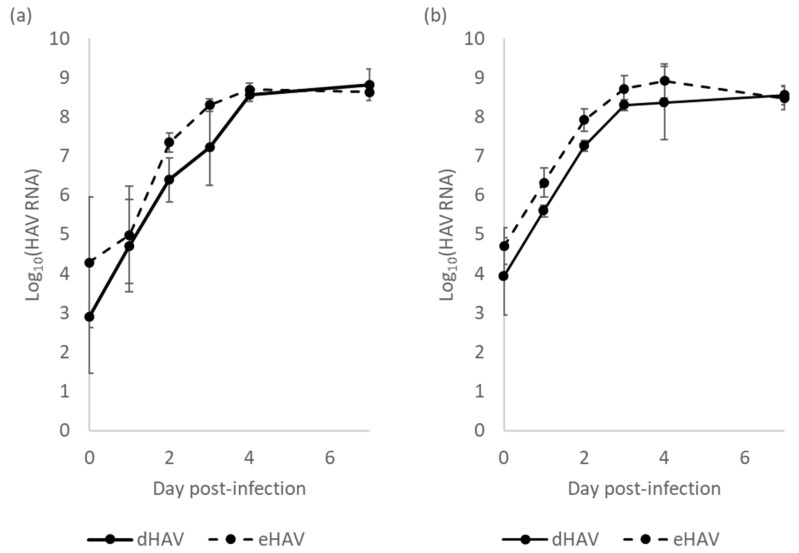
Purified eHAV and nHAV replication in FRhK-4 cells. (**a**) HAV RNA titration in culture supernatants; (**b**) intracellular HAV RNA titration. Day 0 corresponds to day of infection. Results are expressed in log_10_ of genome copies (n = 4).

To compare the replication rates of eHAV and nHAV, FRhK-4 cells were infected with eHAV or nHAV at an M.O.I. of 1. Viral replication within the cells and virus release in the supernatants were assessed up to 7 days post-infection (Figure 4).

As presented in Figure 4a, HAV RNA concentration increased in culture supernatants for both eHAV- and nHAV-infected cells. In eHAV-infected cells, from day 0 to 4, genome quantity increased by 4.4 log_10_ and then reached a stationary phase between day 4 and 7 at 8.64 ± 0.11 log_10_ HAV RNA/mL. In nHAV-infected cells, genome quantity increased by 5.65 log_10_ from day 0 to 4 and reached a stationary phase until day 7 at 8.82 ± 0.18 log_10_ HAV RNA/mL. A significant difference in virus release was observed between both types of viral particles only at day 2 (ANOVA; *p* < 0.01). A similar kinetic pattern was observed for intracellular replication of both eHAV and nHAV (Figure 4b) as the HAV RNA concentration increased by 3.86 log_10_ and 4.21 log_10_ from day 0 to 4 and then stabilized between day 4 and 7 at 7.43 ± 0.07 log_10_ HAV RNA and 7.65 ± 0.14 log_10_ HAV RNA for eHAV and nHAV, respectively. The amounts of the virus within the infected cells were significantly higher at days 1 and 2 following the infection with eHAV in comparison with nHAV (ANOVA; *p* < 0.01). However, no significant differences in the HAV RNA quantity produced during eHAV or nHAV replication were observed at days 4 and 7, indicating that the presence or absence of a quasi-envelope does not impact the amount of HAV produced within FRhK-4 cells nor virus release in the supernatant.

As a whole, the replication of nHAV is as productive as the replication of eHAV, which makes nHAV a good and convenient model of cultivable nHAV.

## 4. Discussion

In this study, an efficient HAV delipidation method was proposed. The 5% Tween-20 and 0.1% 2-MercaptoEthanol mixture showed the best efficiency in removing the lipid bilayer, as seen by sucrose gradient ultracentrifugation experiments and TEM. Even if the viral stock is not entirely delipidated by the chemical treatment, a major part of the lipid bilayers is removed from the viral particles. In addition, the use of additional purification steps following the chemical delipidation treatment (i.e., ultracentrifugation on sucrose gradients and ultrafiltration) leads to only the recovery of naked HAV particles. This method provides an effective way of producing infectious nHAV as a model of cultivable nHAV.

The nHAV infectious stock titres obtained are sufficiently high and close to those of eHAV, enabling the study of nHAV replication in the same conditions as eHAV. The nHAV particles are therefore convenient for further investigations such as inactivation or persistence studies, especially because, due to the life cycle of HAV and its faeco-oral transmission route, the particles’ phenotype that circulates, persists, and contaminates the environment and water corresponds to the naked one. However, the nHAV produced by the described method is single-use. Due to the replication cycle of HAV, in vitro cell infection by nHAV will result in eHAV production because of cellular membrane hijacking [3] during the last step of viral replication, especially virus release from infected cells. Although sui-table, the developed method could be improved or partially automated to make it easier to use and produce larger stocks of nHAV.

Regarding the viral replication cycle, nHAV multiplication seems slightly lower than eHAV between day 2 and 4 following the infection at the same M.O.I. Despite the slight impact during multiplication within cells, no significant difference was observed in virus yields (rate) regardless of the type of HAV particle. Our results suggest that early steps of the HAV life cycle could be impaired. Since eHAV and nHAV are structurally different, virus adsorption on cellular receptors and the mechanisms of virus entry and uncoating within cells and genome uncoating may be different for both types of viral particles [22]. In a previous study, the cellular receptor TIM1 (T cell immunoglobulin mucin receptor 1) was identified to be used in HAV infections, but its implication was recently discussed in HAV cellular entry as it only facilitates the cellular entry of quasi-enveloped HAV particles [5,17]. Gangliosides were shown to be essential for viral capsid uncoating, and this is for both types of particles. Indeed, in the absence of gangliosides, the entry of eHAV and nHAV is successful but viral particles accumulate within LAMP1+ endolysosomes [18]. However, the HAV capsid is excessively stable and the underlying mechanisms of capsid uncoating remain unknown [2]. High-affinity antibodies were shown to destabilize the HAV capsid [18], probably by mimicking an interaction with a specific cellular receptor that was proposed to be expressed in late endolysosomal membranes [22]. Concomitantly with this knowledge, deeper insights into particular HAV capsid integrity still need to be gained.

HAV particles are known to be particularly resistant to extreme conditions such as high pH or temperatures and can persist for a long time in the environment [18,23]. Most of the studies that have demonstrated their resistance were conducted with culture supernatants containing mainly eHAV, as it is the major type of particle available for in vitro use. However, since eHAV and nHAV particles are structurally different, the physicochemical mechanisms involved in the resistance and persistence of both types of viral particles could be different. Even if the main transmission route of HAV is by direct contact through the faecal–oral route, foodborne transmission of HAV can still happen. Several foods were identified to be at risk of foodborne HAV transmission such as seafood, frozen berries, and leafy greens. Foodborne transmission of HAV has become a concern due to the changes in its epidemiological distribution and changes in eating habits, involving more frozen products and imported foods [24,25]. However, several studies were conducted to assess HAV inactivation and persistence in foods [16,26,27] but were performed using infected cell supernatants (containing mainly eHAV particles). Therefore, studies on the resistance and persistence of HAV may be conducted using nHAV, as it is this type of particle that contaminates foods.

This delipidation method using a chemical mixture (5% Tween-20 and 0.1% 2-MercaptoEthanol) followed by purification steps provides a truly efficient way of cultivable nHAV production. As this type of viral particle circulates and persists in the environment, in water, and between individuals, naked HAV particles are found on several foods and thus are a main concern for food safety. Having access to cultivable nHAV is crucial to carrying out experimental studies to assess HAV persistence in food and to validate the efficacy of inactivation processes used in the food industry. Such lab-scale studies are essential to provide guidelines for risk management and better food safety practices.

## Figures and Tables

**Table 1:** Reaction mix composition and concentrations for HAV RNA quantification by RT-qPCR.

Mix RT-qPCR	Final Concentration
**Ultrasense^TM^ reaction mix**	1X
**Forward primer**	500 nM
**Reverse primer**	900 nM
**TaqMan probe**	250 nM
**RNase inhibitor**	20 U/µL
**Bovin Serum Albumin**	1X
**Ultrasense^TM^ enzyme mix**	1.26 µL per sample
**Rnase-free water**	*q.s. (quantum satis)* 20 µL per sample

## Data Availability

The original contributions presented in the study are included in the article, further inquiries can be directed to the corresponding author/s.
